# Advancing antibody-drug conjugates in gynecological malignancies: myth or reality?

**DOI:** 10.37349/etat.2022.00077

**Published:** 2022-04-19

**Authors:** Marta Nerone, Maria Del Grande, Cristiana Sessa, Ilaria Colombo

**Affiliations:** Service of Medical Oncology, Oncology Institute of Southern Switzerland (IOSI), EOC, 6500 Bellinzona, Switzerland; Agostino Gemelli University Policlinic, Italy

**Keywords:** Antibody-drug conjugates, gynecological malignancies, ovarian cancer, endometrial cancer, cervical cancer

## Abstract

Antibody-drug conjugates (ADCs) represent a new class of therapeutic agents designed to target specific antigens on tumor cells, combining the specificity of monoclonal antibodies to the cytotoxicity of classic chemotherapy agents. These drugs have been extensively studied both in solid and hematologic malignancies, leading to substantial improvement in the therapeutic landscape for several tumors. Despite no ADC have been yet approved for the treatment of gynecological malignancies, some agents have shown promising results and might have the potential to become part of the standard of care. Among them, mirvetuximab soravtansine has shown activity in platinum-resistant ovarian cancer with high folate-α receptor expression, as a single agent and in combination. Tisotumab vedotin is active in patients with pre-treated cervical cancer, and further investigation is ongoing. The purpose of this review is to summarize the structural and functional characteristics of ADCs and analyze the most recent and promising data regarding the clinical development of ADCs in gynecological malignancies. The available data on the efficacy of the more studied ADCs in ovarian, endometrial, and cervical cancers will be discussed along with toxicities of special interest, the mechanisms of resistance, and future possible drugs combination.

## Introduction

Antibody-drug conjugates (ADCs) are a relatively recently developed class of drugs with a unique structure consisting of an antibody capable of recognizing a specific cellular antigen, a cytotoxic molecule bounded to this antibody, and a linker that holds the two parts together ensuring the stability of the molecule. These drugs were introduced with the aim of improving chemotherapy efficacy, while limiting systemic toxicity since the specific antigen-antibody interaction aims to directly deliver the cytotoxic agent into the tumor cells, sparing healthy tissue that does not express or only minimally express the targeted antigen [1]. This approach to selectively deliver a cytotoxic agent to the cancer cells (the ‘magic bullet’) was first developed by Paul Ehrlich at the beginning of the twentieth century to overcome the limitation of the narrow therapeutic index of many potent chemotherapy agents [2]. Despite the possibility to directly drive the cytotoxic agent into the cancer cells, ADCs are commonly characterized by different adverse events (AEs). These are mainly due to the expression of the targeted antigen in non-cancer cells and the occurrence of off-target toxicities due to the release of the payload into the bloodstream, influencing the maximum tolerated dose (MTD) of these compounds [3].

The first ADC that has been used in clinical practice is gemtuzumab ozogamicin for acute myeloid leukemia, approved by the Food and Drug Administration (FDA) in 2001 [4], followed by brentuximab vedotin in Hodgkin lymphoma [5]. The first FDA-approved ADC in solid malignancies is trastuzumab emtansine (T-DM1) for the treatment of metastatic and early-stage breast cancer [6, 7]. Since then, this field has been rapidly evolving with currently > 100 different ADCs under investigation across solid and hematological malignancies. Four ADCs are available in clinical practice for the treatment of patients with solid tumors: T-DM1 and trastuzumab deruxtecan (T-DXd) for human epidermal growth factor 2 (HER2)-positive breast cancer [8], sacituzumab govitecan for triple-negative breast cancer [9], and enfortumab vedotin for metastatic urothelial carcinoma [10].

Different ADCs are under investigation for the treatment of gynecological malignancies and some with promising results. The aim of this review is to describe the state of the art of ADCs in gynecological malignancies at different stages of research and development.

## Structure and mechanism of action of ADCs

### Key components of ADCs

Antibody/antigen: The first step in the development of an ADC is the selection of the antibody, which must be specific to a defined antigen and have low immunogenicity [11]. Most of the antibodies from which the new generation ADCs are formed are fully humanized, typically immunoglobulin G (IgG). The introduction of fully humanized antibodies has been paramount to reduce the immunogenicity of the murine and the chimeric (mouse/human) first- and second-generation ADCs [12]. The antigen must be present on the cell surface and be expressed exclusively, or at least preferentially, in tumor cells as opposed to normal tissue, to limit the off-target systemic toxicities [1]. The antigen-antibody binding on the cancer cell surface is followed by internalization of the ADC with subsequent lysosomal degradation and intracellular release of the cytotoxic agents, a process that leads to cell death [13]. The amount of ADC that is internalized depends on the antigen density on the cell surface [14]. However, heterogenous results are available on the correlation between the level of the antigen expression and the ADC’s efficacy [15, 16].

**Figure 1.**
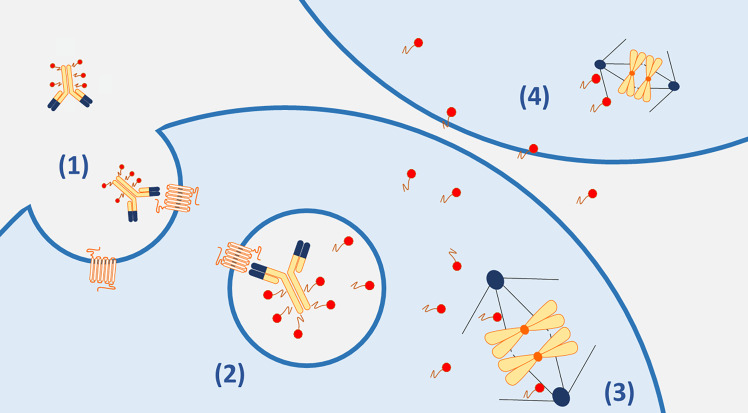
Main mechanisms of action of ADCs. (1). The ADC complex binds to the target antigen on the cancer cell membrane and is internalized; (2). in the lysosome, the payloads are released through linkers cleavage or antibody degradation (in case of non-cleavable linkers); (3). the cytotoxic payloads cause drug-specific microtubule inhibition; (4). the diffusion of cytotoxic payloads across the cell membranes can result in the death of neighboring antigen negative cells (bystander effect)

Payloads: Payloads are extremely small cytotoxic molecules, in the nanomolar or picomolar range, bounded to the antibody structure via a linker. Over the years, several classes of payloads have been developed, but the most employed are auristatins and maytansinoids [17]. Auristatins are synthetic analogs of dolostatin 10, which is a natural antimitotic that caused inhibition of tubulin assembly. Among auristatins, the most widely used is monomethyl auristatine E (MMAE, vedotin) [18]. The other group of commonly used payloads is the maytansinoids, synthetic analogs of maytansine that act similarly to the vinca alkaloids inhibiting microtubule assembly. Mertansine (DM1) maytansinoids include emtansine and mertansine; ravtansine maytansinoids include soravtansine and ravtansine (DM4) [19]. The most used payloads in ovarian cancer are MMAE (vedotin) and DM4, both potent microtubule inhibitors. In addition to the direct intracellular effect, MMAE is also able to permeate the cell membrane, ensuring that the cytotoxic molecule spreads from target cells to neighboring cells, exerting its cytotoxic effect even in those cells that do not express the cellular antigen on their surface: this is known as the “bystander effect” [20] (Figure 1).

Other payloads developed in clinical practice are calicheamicin, duocarmicins, and pyrrolobenzodiazepines, which are potent inhibitors of nucleic acid synthesis because of their ability to recognize and bind to specific sequences of the DNA minor groove. SN-38, the active metabolite of irinotecan that acts inhibiting the DNA topoisomerase-I, has also been used as a payload. Due to its potent activity and the consequent toxicity, SN-38 cannot be administered as a free drug. Therefore, ADCs containing SN-38 have been developed, such as sacituzumab govitecan and T-DXd, both of which have already been approved by the FDA for the treatment of metastatic breast cancer [8, 9].

Each ADC is characterized by a specific drug-to-antibody ratio (DAR) [21]. A higher number of cytotoxic molecules (hence a higher DAR) confers greater cytotoxicity, as opposed to an antibody that can bind fewer payloads. However, a high number of payloads may alter the structure of the ADC, compromising its stability, reducing the antigen affinity, or affecting its distribution in the tumor microenvironment [22, 23].

Linker: The antibody portion of an ADC and the cytotoxic warheads are connected through a linker, which influences the stability of the ADC in the bloodstream. The characteristics of the linker impact also the ability to release the cytotoxic payload when the ADC is bounded to the antigen or internalized in the cancer cell, avoiding a premature release, which can lead to an increased incidence of off-target toxicities [24]. Linkers are divided into two main categories: cleavable and non-cleavable. Cleavable linkers can be degraded by protease, acid pH, endosome, or lysosome reactions, thus part of the cytotoxic payloads might be released into the tumor microenvironment, affecting both antigen-expressing targets cells but also non-antigen-expressing surrounding cells through the “bystander effect” [20]. Non-cleavable linkers need the lysosomal proteolytic activity of the antibody to release the payload. The product of this cleavage contains the payload still attached to the linker and this can affect its electrical charge, hydrophobicity, or hydrophilicity. Non-cleavable linkers impact the ability of the payload to cross the membrane and on the other hand, the presence of the linker portion can cause the linker-payload complex to be eliminated from the cell via efflux pumps compromising the intracellular concentration of the payload and leading to drug resistance [25].

### Mechanism of action of ADCs

The main mechanism of action of ADCs relies on the internalization of the cytotoxic payload following the antibody binding on the cell surface target and the subsequent linker breakdown (Figure 1). However, the therapeutic effect of ADCs is exerted through different and complex processes [26]. After administration of an ADC, the conjugate antibody but also the naked antibody and the free payload, are found in the bloodstream and they might induce antitumor activity on their own. The monoclonal antibody part of an ADC, particularly the antigen-binding fragment (Fab) can induce antitumor activity after target engagement even before the cytotoxic payload is released into the cancer cell and this is of relevance for ADCs targeting oncogenic antigens as described for T-DM1 and T-DXd [27, 28]. Moreover, as well known for many monoclonal antibodies, the Fc component of an ADCs can recruit immune effector cells and elicit cancer cells killings via antibody-dependent cell cytotoxicity (ADCC), complement-dependent cytotoxicity (CDC), or antibody-dependent cellular phagocytosis (ADCP), thus acting as a form of immunotherapy [27, 29].

Upon target recognition, the ADC-antigen complex is internalized into the cell via receptor-mediated endocytosis or antigen-independent pinocytosis. Therefore, the efficacy of the ADC might be influenced by the antigen-binding affinity and degree of target internalization [30]. Subsequently, the lysosomes and endosomes activity is necessary to release the free cytotoxic warhead into the cytoplasm, inducing apoptosis and cell death [31]. Ultimately, membrane-permeable payloads can result in cytotoxic activity in the neighboring cells regardless of the expression of the target antigen. This “bystander effect” has a crucial role in the efficacy of ADCs and depends also on linker properties as described above [32].

## ADCs in gynecological malignancies

Despite the significant progress achieved in the treatment of different malignancies, few new therapeutic targets are available for women with gynecological cancer. This has unfortunately led to little improvement over the years in the survival rates of patients with ovarian, endometrial, or cervical cancer. Thus, there is still an unmet need to discover new treatment options to improve patients’ outcomes.

**Figure 2.**
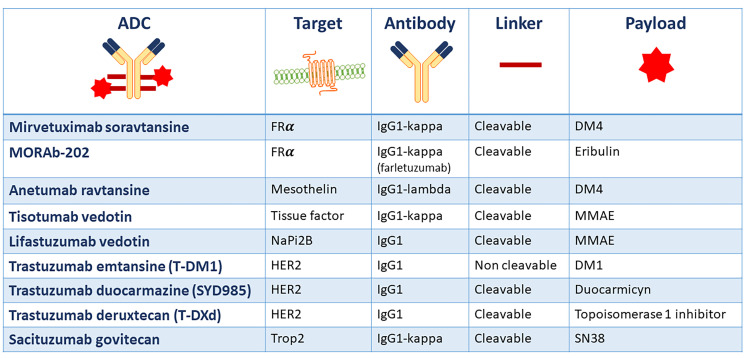
Main ADCs under development in gynecological cancers and their structural composition. FRα: folate receptor alfa; NaPi2b: anti-sodium-dependent phosphate transport protein 2b; Trop2: human trophoblast cell-surface marker 2

Currently, there are no ADCs approved for the treatment of gynecological malignancies, but several trials have shown promising results sustaining the ongoing clinical research to further develop these agents. The composition of the main ADCs under development in gynecological cancers is summarized in Figure 2.

### ADCs in ovarian cancer

The standard of care for advanced ovarian cancer is optimal cytoreductive surgery combined with platinum-based chemotherapy. Maintenance treatment with the antiangiogenic agent bevacizumab and/or poly(ADP-ribose)polymerase (PARP) inhibitors have significantly improved patients’ outcomes and are now part of the standard treatment strategy in first-line setting [33–36]. Despite the initial high response rate, ~80% of patients will eventually experience disease recurrence and progressive development of chemoresistance. In a platinum-resistant setting, treatment options are limited and the prognosis is poor. Available standard treatments are associated with low response rates (15–20%) and limited progression-free survival (PFS; 3–4 months) and overall survival (OS; 12 months) [37, 38]. Furthermore, in the recurrent disease setting, therapeutic options are often limited by the cumulative residual toxicity from previous treatments. The introduction of ADCs might be a valuable opportunity to increase the chemotherapy efficacy, while at the same time minimizing systemic toxicities.

Different cellular surface antigens have been identified in ovarian cancer as possible targets for ADCs with the FRα and mesothelin representing the most investigated [39, 40].

The FRα is a transmembrane protein present on the cell surface that mediates the transport of folate into the cells. This protein is poorly expressed in normal tissue, while a high surface expression has been demonstrated in ovarian, endometrial, breast, and non-small cell lung cancer cells [41, 42]. The increased expression of FRα on tumor cells occurs as a response to an increased demand for folate to sustain tumoral cell growth and proliferation [43]. Notably, FRα expression is a prognostic biomarker in ovarian cancer and its expression correlates with poor response to chemotherapy and worse PFS and OS [44].

Mesothelin is a membrane glycoprotein characterized by minimal expression in normal tissues (limited to mesothelial cells of the pleura, pericardium, and peritoneum) and overexpression in mesotheliomas, ovarian, pancreatic, lung, gastric, and triple-negative breast cancers. Mesothelin promotes tumorigenesis through induction of interleukine-6 (IL-6) and induces resistance to tumor necrosis factor-alpha (TNF-α) induced apoptosis [45]. Notably, high mesothelin expression correlates with poor prognosis in ovarian cancer [46].

The main results from clinical trials investigating an ADC in ovarian cancer are summarized in Table 1. Ongoing clinical trials are reported in Table 2.

**Table 1.** Main clinical trials of ADCs in ovarian cancer

**Target**	**ADC**	**Trial**	**Phase**	**Setting**	**Treatment**	**Primary endpoint**	**Results**
FRα	Mirvetuximab soravtansine	FORWARD I [47] (NCT02631876)	III	Platinum-resistant FRα positive	Mirvetuximab *vs.* chemotherapy of investigator’s choice	PFS	ORR: 24 *vs.* 10% (*P* = 0.014 ) PFS: 4.1 *vs.* 4.4 months (HR 0.98)
		FORWARD II [48–51] (NCT02606305)	Ib/II	Platinum-sensitive FRα positive	Mirvetuximab soravtansine + carboplatin	Safety (phase Ib) ORR (phase II)	ORR: 71% PFS: 15 months
				Platinum-resistant FRα positive	Mirvetuximab soravtansine + pembrolizumab	Safety (phase Ib) ORR (phase II)	ORR: 43% PFS: 5.2 months
				Platinum-resistant FRα positive	Mirvetuximab soravtansine + bevacizumab	Safety (phase Ib) ORR (phase II)	ORR: 39% PFS: 6.9 months
				Platinum-resistant and sensitive FRα positive	Mirvetuximab soravtansine + bevacizumab	Safety (phase Ib) ORR (phase II)	ORR: 50% PFS: 8.3 months
	MORAb-202	NCT03386942 [52]	I	Platinum-resistant FRα positive	Farletuzumab conjugated with eribuline	DLTs	ORR: 37.5%
Mesothelin	Anetumab ravtansine	NCT01439152 [53]	I	Platinum-resistant and partially platinum sensitive	Anetumab ravtansine	DLTs	ORR: 9%
	DMOT4039A (RG7600)	NCT01469793 [54]	I	Platinum-resistant	DMOT4039A	DLTs/RP2D	ORR: 30%
	BMS-986148	CA008-008 [55] (NCT02341625)	I/IIa	Platinum unselected	BMS-986148	Safety	ORR: 10%
TF	Tisotumab vedotin	InnovaTV 201 [56] (NCT02001623)	I/II	Advanced solid tumors including ovarian cancer platinum unselected	Tisotumab vedotin	Safety	ORR: 13.9%
MUC16	DMUC4064A	NCT02146313 [57]	I	Platinum-resistant	DMUC4064A	Safety	ORR: 25%
NaPi2B	Lifastuzumab vedotin	NCT01363947 [58]	I	Platinum-resistant	Lifastuzumab vedotin	Safety	ORR: 36.7%
		NCT01991210 [59]	II	Platinum-resistant	Lifastuzumab vedotin *vs.* PLD	PFS	ORR: 34 *vs.* 15% PFS: 5.3 *vs.* 3.1 months (HR 0.78)

MUC16: mucine 16; PLD: pegylated liposomal doxorubicin; RP2D: recommended phase 2 dose; ORR: overall response rate; HR: hazard ratio; *vs.*: *versus*; DLTs: dose-limiting toxicities; TF: tissue factor

**Table 2.** Main ongoing clinical trials of ADCs in ovarian cancer

**Target**	**ADC**	**Trial**	**Phase**	**Setting**	**Treatment**	**Primary endpoint**
FRα	Mirvetuximab soravtansine	MIROVA (NCT0427442)	II	Platinum-eligible FRα positive	Mirvetuximab soravtansine + carboplatin *vs.* platinum-based chemotherapy	PFS
		MIRASOL (NCT04209855)	III	Platinum-resistant FRα positive high	Mirvetuximab soravtansine *vs.* chemotherapy of investigator’s choice	PFS
		SORAYA (NCT04296890)	III	Platinum-resistant FRα positive high	Mirvetuximab soravtansine	ORR
		NCT03552471	I	Platinum-resistant and *BRCA* mutated platinum-sensitive FRα positive	Mirvetuximab soravtansine + rucaparib	RP2D
		NCT02996825	I	Platinum-resistant FRα positive	Mirvetuximab soravtansine +gemcitabine	RP2D
		NCT04606914	II	Neoadjuvant, newly diagnosed FRα positive high	Carboplatin + mirvetuximab soravtansine	PFS
Mesothelin	Anetumab ravtansine	NCT02751918	Ib	Platinum-resistant	Anetumab ravtansine + PLD	MTD
		NCT03587311	II	Platinum-resistant	Bevacizumab + anetumab ravtansine or paclitaxel	PFS
TF	Tisotumab vedotin	InnovaTV 208 (NCT03657043)	II	Platinum-resistant	Tisotumab vedotin	ORR

*BRCA*: breast cancer gene

#### Anti-folate receptor α: mirvetuximab soravtansine

Mirvetuximab soravtansine is an anti-FRα ADC, formed by the humanized IgG1 antibody conjugated to a DM4 payload via a cleavable linker. Structurally, DM4 is conjugated to the antibody with a DAR of 3.5:1. The final payload metabolite inhibits tubulin and causes cell cycle arrest in the G2–M phase, ultimately causing cell death. DM4 is electrically neutral and lipophilic, thus capable of crossing cell membranes and causing the “bystander effect” [20]. Mirvetuximab soravtansine is one of the first ADC investigated in ovarian cancer, and it is the only one for which results from a phase III study are available to date. A phase I dose-escalation trial in solid tumors, including patients with pre-treated epithelial ovarian cancer (EOC), established the RP2D at 6 mg/kg every 3 weeks and preliminary signs of activity were observed [60]. The DLTs observed in this trial were grade (G) 3 hypophosphatemia and G3 punctate keratitis [60]. A subsequent study was then conducted in an expansion cohort of 46 patients with platinum-resistant EOC and FRα positivity assessed on immunohistochemistry (defined as ≥ 25% tumor cells with at least 2+ staining). The ORR in this study was 26%, including one complete response (CR) and 11 partial responses (PRs), with a median PFS (mPFS) of 4.8 months and a median duration of response (DoR) of 19.1 weeks [15]. A phase Ib study confirmed the correlation between the level of FRα expression and mirvetuximab soravtansine efficacy [61]. No objective response was observed in low expressors (25% to 49% of tumor cells with ≥ 2+ staining intensity), with a mPFS of 2.8 months, while in medium expressors (50% to 74% of tumor cells with ≥ 2+ staining intensity), ORR was 20% with a mPFS of 3.9 months. In high-expressors (≥ 75% tumor cells with ≥ 2+ staining intensity), ORR was 31% with a mPFS of 5.4 months [61]. Given the evidence of increased benefit in patients with medium or high FRα expression, a subsequent phase III study was designed incorporating the same immunohistochemical threshold.

FORWARD I is a phase III clinical trial that enrolled 366 women with platinum-resistant EOC with medium or high FRα expression and pre-treated with 1–3 lines of chemotherapy [47]. The FRα threshold for positivity by immunohistochemistry was defined as > 50% of tumor cells with any FRα membrane staining visible at 10 microscope objective with a value of 50–74% and ≥ 75% representing medium and high expression, respectively. Patients were randomized 2:1 to receive mirvetuximab soravtansine 6 mg/kg every 3 weeks *versus* investigator’s choice chemotherapy (weekly paclitaxel, PLD, or topotecan). A higher ORR was observed in patients treated with mirvetuximab soravtansine compared to chemotherapy (24 *versus* 10%, *P* = 0.014), with no significant improvement in PFS (4.1 months *versus* 4.4 months, HR = 0.98) and in OS (HR = 0.62) [47]. The most common all grades AEs reported in these trials included nausea (45.7% of patients), blurred vision (42%), keratopathy (32.5%) diarrhea (31.3%), fatigue (28.8%) and peripheral neuropathy (26.7%) [47]. An AE of special interest was the ocular toxicity, characterized by blurred vision, dry eyes, or corneal abnormalities, which could be managed with topical steroids and/or with dose reduction [47, 60, 62]. Pneumonitis is another AE of special interest and occurred in 2.9% of enrolled patients (G1–3) [47, 60].

To evaluate the potential efficacy of mirvetuximab soravtansine in a better-selected population, two subsequent studies in patients with platinum-resistant EOC and high FRα expression (defined as ≥ 75% of cells with at least a score 2 staining intensity) have been initiated. First, the single-arm SORAYA trial (NCT04296890; prior bevacizumab required), aimed at supporting accelerated approval, with ORR as the primary endpoint. Secondly, the randomized confirmatory phase III MIRASOL trial (NCT04209855) comparing mirvetuximab soravtansine monotherapy with investigator’s choice chemotherapy, is still ongoing and results are eagerly awaited.

Given the limited activity as a single agent, different combinations with chemotherapy, antiangiogenic agents, or immune checkpoint inhibitors have been explored. FORWARD II is a phase Ib/II trial evaluating the efficacy of mirvetuximab soravtansine in combination with bevacizumab, carboplatin, PLD, or pembrolizumab. In the cohort of platinum-sensitive EOC, FRα positive (≥ 25% tumor cells with at least 2+ staining) patients were treated with carboplatin (AUC5) and mirvetuximab soravtansine 6 mg/kg for six cycles followed by maintenance treatment with mirvetuximab soravtansine. In this study, the highest benefit was seen in patients with a medium to high FRα expression, with an ORR of 80% [48]. Preliminary data combining mirvetuximab soravtansine and pembrolizumab in patients with platinum-resistant EOC, showed an ORR of 43% with a mPFS of 5.2 months and a median DoR of 30.1 months, with higher PFS and DoR in medium-high FRα expressors [49]. In patients with the platinum-resistant disease, the combination of mirvetuximab soravtansine and bevacizumab achieved an ORR of 39% with a mPFS of 6.9 months and a median DoR of 8.6 months. In the bevacizumab-naive population the efficacy was higher (ORR 56%, PFS 9.9 months, and median DoR of 12 months). Overall, the combination regimen was well tolerated [50]. At the 2021 Annual Meeting of the American Society of Clinical Oncology (ASCO), new data on the combination of mirvetuximab and bevacizumab in a platinum agnostic population of patients with recurrent EOC were presented [51]. Patients with high FRα expression had significantly higher mPFS (10.6 months) compared to patients with medium FRα expression (5.4 months). Similarly, patients with platinum-sensitive ovarian cancer achieved a better ORR and mPFS compared to platinum-resistant ovarian cancer (PFS: 13.3 months *versus* 9.7 months). Notably, in patients with high FRα expression, ORR was 64% and median DOR 11.8 months, and these outcomes were achieved irrespective of platinum sensitivity [51].

Interestingly, an expansion cohort of the FORWARD II study is still ongoing to evaluate the triple combination of mirvetuximab soravtansine, carboplatin, and bevacizumab, in patients with platinum-sensitive recurrent EOC. Both mirvetuximab soravtansine and bevacizumab were continued as maintenance therapy after the six combination cycles. In this group of patients, the ORR was 80% and PFS data were still immature at the time of data presentation. G3 AEs included thrombocytopenia, neutropenia, hypertension, diarrhea, nausea, and fatigue [63].

Taken together, this data suggests that mirvetuximab soravtansine exhibits a higher activity in high FRα expressors and when is used in combination with other chemotherapy or targeted agents, including PARP inhibitors (NCT03552471). Nevertheless, many open questions still remain, particularly on the role of FRα expression when mirvetuximab soravtansine is used in combination with other agents and which predictive biomarkers of response might support a better patient selection.

Other anti-FRα ADCs are also under investigation in phase I trials, including STRO-002 [64] and MORAb-202 [52] which showed promising results in FRα-expressing solid tumors, including ovarian and endometrial neoplasms.

#### Anti-mesothelin: anetumab ravtansine

Anetumab ravtansine is an anti-mesothelin ADC composed of an IgG1 antibody conjugated to the DM4 payload. It has a DAR of 3.2 with a charged, cleavable linker capable of bystander effect also on adjacent mesothelin-negative cells [65]. A phase I dose-escalation trial included 45 patients (4 with EOC) and defined the MTD at 6.5 mg/kg every 3 weeks, which was then used in the expansion cohort of the same trial that enrolled 21 patients with EOC. Among patients with ovarian cancer who received the MTD, 9% had a PR and 50% stable disease (SD) [53]. The treatment was well tolerated, with any grade fatigue (57.8% of all patients), nausea (50.5%), diarrhea (41.3%), anorexia (34%), vomiting (27.5%), peripheral sensory neuropathy (25.7%), asymptomatic aspartate aminotransferase (AST) increase (29.4%), blurred vision (22%) and keratitis (19%) being the main AEs, as also seen with other ADCs containing the ravtansine maytansinoid [53]. The reported DLTs were G3 AST increase, G3 hypertension, G3 hyponatremia, G3 peripheral neuropathy, G4 keratitis, and G4 amylase and lipase increase.

Anetumab ravtansine has also been evaluated in combination with other agents. A phase Ib study in patients with platinum-resistant EOC investigated the combination of anetumab ravtansine and PLD, showing a PR in 52% of patients (lasting > 250 days in 6) and SD in 33% [66]. A randomized phase II trial of anetumab ravtansine plus bevacizumab *versus* paclitaxel plus bevacizumab in patients with platinum-resistant or refractory EOC is ongoing (NCT03587311).

#### Other ADCs in ovarian cancer

Other ADCs have been developed and are under preclinical and clinical evaluation in ovarian cancer. These ADCs have been directed against specific antigens expressed in several solid tumors including ovarian cancer, among which the most investigated are cancer antigen 125 (CA125 or MUC16) [57, 67, 68], NaPi2b [58, 59, 69–71], Trop2 [72], TF [73–75], protein tyrosine kinase 7 (PTK7) [76], CD166 and Notch3 [77].

### ADCs in endometrial cancer

Endometrial carcinoma is the most frequent gynecological neoplasm in western countries, and unlike ovarian and cervical cancer, its incidence is increasing [78]. The prognosis of this disease depends on the stage at diagnosis, histological subtype, grade, presence of lympho-vascular space invasion, and molecular characteristics [79]. Women diagnosed with early-stage well-differentiated endometrioid carcinoma have a 5-year survival rate of 90%. However, ~35% of endometrial cancer has more aggressive histology (poorly differentiated endometrioid, serous or clear cell carcinoma) which is more frequently present at stage III and IV and are poorly responsive to standard treatment [79]. Despite the recent advancement in the understanding of endometrial cancer biology and the introduction of novel treatment strategies, such as immunotherapy for microsatellite instable and a combination of immunotherapy and antiangiogenic agents for microsatellite stable tumors, the prognosis of patients with advanced or recurrent endometrial cancer remains poor [80–82]. Thus, the identification of new treatment options is paramount to improving patients’ outcomes.

In endometrial cancer, contrary to what is described in ovarian cancer, the field of ADCs is still in an early phase of development. However, therapeutic targets of potential interest have been identified and preliminary results are available (Table 3). Furthermore, different trials are ongoing to further explore the safety and efficacy of ADCs in this malignancy (Table 4).

**Table 3.** Main clinical trials of ADCs in endometrial and cervical cancer

**Target**	**ADC**	**Trial**	**Phase**	**Setting**	**Treatment**	**Primary endpoint**	**Results**
HER2	T-DM1	NCT02675829 [83]	II	HER2 pos tumors including endometrial cancer	T-DM1	ORR	ORR: 22%
		NCT02465060 [84]	II	HER2 pos including endometrial and ovarian cancer	T-DM1	ORR	ORR: 9%
FRα	Mirvetuximab soravtansine	NCT01609556 [60]	I	FRα pos tumors including metastatic endometrial cancer	Mirvetuximab soravtansine	MTD/RP2D	ORR: 0%
Trop2	Sacituzumab govitecan	IMMU-132-01 (NCT01631552) [85]	I/II	Advanced epithelial cancer including endometrial, cervical and ovarian cancer	Sacituzumab govitecan	Safety (phase I) ORR (phase II)	ORR: 22.2%
TF	Tisotumab vedotin	InnovaTV 204 (NCT03438396) [86]	II	Previously treated recurrent or metastatic cervical cancer	Tisotumab vedotin	ORR	ORR: 24% PFS: 4.2 months
		InnovaTV 205 (NCT03786081) [87]	I/II	Recurrent or metastatic cervical cancer, first line	Tisotumab vedotin + carboplatin	DLT (phase I) ORR (phase II)	ORR: 55% PFS: 9.5 months
				Recurrent or metastatic cervical cancer, second or third line	Tisotumab vedotin + pembrolizumab	DLT (phase I) ORR (phase II)	ORR: 38% PFS: 5.6 months

**Table 4.** Main ongoing clinical trials of ADCs in endometrial and cervical cancer

**Target**	**ADC**	**Trial**	**Phase**	**Setting**	**Treatment**	**Primary endpoint**
HER2	Trastuzumab duocarmazine (SYD985)	NCT04205630	II	HER2 pos metastatic endometrial cancer	Trastuzumab duocarmazine	ORR
	T-DXd	NCT04482309	II	HER2 pos tumors including ovarian, endometrial, and cervical cancer	T-DXd	ORR
FRα	Mirvetuximab soravtansine	NCT03832361	II	FRα pos persistent or recurrent endometrial cancer	Mirvetuximab soravtansine	ORR
		NCT03835819	II	FRα pos advanced or recurrent serous endometrial cancer	Mirvetuximab soravtansine + pembrolizumab	ORR/PFS
Trop2	Sacituzumab govitecan	NCT04251416	II	Trop2 pos persistent or recurrent endometrial cancer	Sacituzumab govitecan	ORR
TF	Tisotumab vedotin	InnovaTV 301 (NCT04697628)	III	Previously treated recurrent or metastatic cervical cancer	Tisotumab vedotin *vs.* chemotherapy	OS

#### Anti-epidermal growth factor receptor-2: T-DM1, trastuzumab duocarmazine, and T-DXd

HER2 is overexpressed in up to 35% of cases of endometrial cancer, particularly in the more aggressive histological subtypes, such as serous and carcinosarcoma [88]. This has supported the investigation of HER2 targeting agents in these aggressive gynecologic malignancies.

A phase II randomized study of trastuzumab in combination with platinum-based chemotherapy has been conducted in patients with HER2/neu overexpressing uterine serous carcinoma. The combination of trastuzumab with carboplatin-paclitaxel showed an improvement in PFS when used in first or subsequent lines of treatment (12.6 months *versus* 8 months, HR = 0.44 in the overall study population) [89].

Beyond trastuzumab, several preclinical studies evaluated the efficacy of the ADC T-DM1 in HER2-amplified ovarian and uterine carcinosarcomas, both on cell lines and xenografts, showing a greater antitumor efficacy compared to single-agent trastuzumab [90, 91] and to the combination of trastuzumab and pertuzumab [92].

Two small clinical trials have evaluated the efficacy of T-DM1 in patients with different types of HER2 amplified malignancies [83, 84]. A basket trial enrolled 58 patients with HER2 amplified advanced solid tumors (lung, endometrial, salivary gland, biliary tract, ovarian, bladder, colorectal, and other cancers) and were treated with T-DM1 3.6 mg/kg i.v. once every three weeks. The ORR was 26% (14/53), 22% (4/18), and 17% (1/6) in the overall population, endometrial and ovarian cancer, respectively [83]. Another study enrolled 38 patients with HER2 amplified tumors other than breast and gastric/gastroesophageal junction adenocarcinomas, to receive T-DM1. Fourteen patients were enrolled, including serous EOC, mixed serous and endometrioid endometrial adenocarcinoma, serous endometrial adenocarcinoma, carcinosarcoma, and mucinous adenocarcinoma of the cervix [84]. Eight out of ten patients with endometrial or ovarian cancer achieved an SD and in the overall study population, the median DoR was 4.6 months [84].

Another ADC targeting HER2, trastuzumab duocarmazine (SYD985), has been investigated in endometrial cancer [93]. Trastuzumab duocarmazine is an ADC composed of trastuzumab linked to the toxic payload duocarmycin via a cleavable linker. The linker cleavage by tumor proteases and the release of the membrane-permeable active toxin causes cell killing not only of the HER2 positive cells but also of the neighboring non-antigen-expressing tumor cells through the “bystander killing effect” [93]. This mechanism of action differs from T-DM1, in which the antibody-drug link is not cleavable and as a consequence, the “bystander killing effect” is less relevant. *In vitro* and *in vivo* studies demonstrated the higher antitumor potency of trastuzumab duocarmazine, both in carcinosarcoma and EOC cell lines and xenografts [94]. This compound is currently being evaluated as a single agent in phase II clinical trial in patients with HER2- positive endometrial cancer progressing after first-line platinum-based chemotherapy (NCT04205630).

T-DXd is another anti-HER2 ADC conjugated to a potent topoisomerase 1 inhibitor, that has shown significant and durable efficacy in patients with HER2 positive breast [8, 95] and gastric cancer [96] and has the potential to also improve the outcome of patients with other HER2 positive solid tumors. Different trials are ongoing to confirm its activity even in gynecological malignancies (NCT04482309).

#### Anti-folate receptor α: mirvetuximab soravtansine

The FRα has been identified as a potential therapeutic target also in endometrial cancer [97]. The overexpression of FRα has been associated with worse outcomes and co-occurs with other poor prognostic factors including advanced stage, non-endometrioid histology, and high grade.

A phase I dose-escalation study of mirvetuximab soravtansine in patients with solid tumors not selected for FRα expression, enrolled a total of 44 subjects including 11 with serous or endometrioid endometrial cancer [60]. The primary objectives of the study were to determine the MTD (not reached) and the RP2D, which was established at 6.0 mg/kg once every three weeks. Two patients with endometrial cancer achieved a clinical benefit: SD lasting more than 4 weeks in one patient and a CA125 response in the other [60]. These data encouraged the development of an ongoing phase II study evaluating the activity and safety profile of mirvetuximab soravtansine in patients with persistent or recurrent endometrial cancer overexpressing FRα (NCT03832361). Eligible tumor histology includes serous, G2–G3 endometroid endometrial carcinoma or carcinosarcoma with high grade serous or G2–G3 endometrioid components.

#### Anti-human trophoblast cell-surface marker: sacituzumab govitecan

Sacituzumab govitecan is a humanized anti-Trop2 antibody, conjugated to the active metabolite of irinotecan (SN-38) via a cleavable linker. Following the positive results achieved in breast and bladder [9, 98] cancers, this agent is now under investigation in other solid tumors, including serous and endometroid endometrial cancer.

The cellular target of this drug is the Trop2, a transmembrane glycoprotein originally identified in human placental tissue and subsequently found to be highly expressed in various type of epithelial tumors. Preclinical studies have shown that Trop2 promotes cell proliferation, inhibits apoptosis, accelerates cell cycle progression and favors tissue invasion and metastasis [99]. Tissue overexpression of Trop2 is also an independent marker of poor prognosis in several neoplasms, including endometrial cancer [100, 101]. The low Trop2 expression in healthy tissues makes it a suitable target for the development of ADCs. SN-38 molecules are bound to the antibody by a cleavable linker with a high DAR (8:1) without a negative effect on pharmacokinetics [72]. The cleavable linker ensures that the cytotoxic molecule is also effective on the neighboring Trop2 negative cells through the “bystander effect”. This mechanism is of particular relevance in tumors with heterogeneous surface expression of Trop2, as it has been observed in endometrial cancer [102]. Preclinical studies demonstrated the *in vitro* and *in vivo* efficacy of sacituzumab govitecan in Trop2 positive endometrioid carcinoma cell lines and xenografts [100, 103].

In the phase I/II basket trial of single-agent sacituzumab govitecan in patients with epithelial cancer (NCT01631552), 18 women with advanced/recurrent endometrial carcinoma were included (histology unspecified) and determination of Trop2 expression was not required [85]. The most common treatment-related AEs were nausea (62.6%), diarrhea (56.2%), fatigue (48.3%), alopecia (40.4%), and neutropenia (57.8%), consistently with the toxicity profile of the irinotecan-derived payload. Clinical activity was seen across tumors subtypes. In the endometrial cohort, the ORR was 22%, mPFS was 3.2 months [confidence interval (CI): 1.9–9.4], and median OS 11.9 months [85].

A phase II study of single-agent sacituzumab govitecan in patients with persistent or recurrent endometrial cancer, who have failed at least a prior platinum-based chemotherapy or refractory to platinum-based chemotherapy, is ongoing (NCT04251416). In this trial, elevated Trop2 expression (at least 2+) is required for patients’ selection.

### ADCs in cervical cancer

Despite the ongoing progress made with the human papillomavirus (HPV) vaccination, cervical cancer remains the fourth most frequent and fatal cancer among women [104]. The current standard of care in relapsed or metastatic disease is the combination of paclitaxel, platinum, and bevacizumab, following the results of a clinical trial showing a survival benefit with the addition of bevacizumab to chemotherapy in this setting [105]. The therapeutic landscape for this disease is rapidly evolving with the incorporation of immune checkpoint inhibitors. The KEYNOTE-826 trial showed a PFS and OS benefit for patients treated in first line with the anti-programmed death 1 (PD-1) pembrolizumab in addition to platinum and paclitaxel +/− bevacizumab, regardless of programmed death-ligand 1 (PD-L1) expression [106]. When progressing after first-line, there is no effective standard of care treatment in cervical cancer and the available therapeutic options have shown little benefit with low survival rates and ORR (15% pemetrexed [107], 14% vinorelbine [108], 5% gemcitabine [109], 11% bevacizumab [110], 14% pembrolizumab [111]). A phase III trial investigating the anti-PD-1 agent cemiplimab *versus* single-agent chemotherapy of physician’s choice, resulted in an improvement in median OS (12 months *versus* 8.5 months, HR 0.69) and ORR (16.4 *versus* 6.3%) and similar mPFS (2.8 months *versus* 2.9 months) [112].

Thus, the use of drugs with alternative targets and different mechanisms of action is an important clinical need in patients with relapsed or metastatic cervical cancer after failure of first-line standard treatment.

Two main cellular antigens have been identified as possible targets for ADCs in cervical cancer.

The TF is a transmembrane protein involved in the extrinsic pathway of the coagulation cascade, but also in angiogenesis, cell adhesion, mobility, and cell survival [113]. It is highly prevalent in many solid tumors, including cervical and uterine cancer, and it is not expressed in endometrial or normal cervical tissue, representing an ideal therapeutic target.

Another promising therapeutic target, already described in endometrial cancer, is the Trop2, whose expression has been also evaluated in cervical neoplasms [114].

#### Anti-tissue factor: tisotumab vedotin

Tisotumab vedotin is an ADC consisting of a fully human monoclonal antibody conjugated to the microtubule inhibitor MMAE through a cleavable linker. Tisotumab vedotin releases MMAE into TF-expressing cells causing direct cytotoxicity and a “bystander killing effect” in neighboring cells that do not express TF or express it heterogeneously.

In the multicentre, single-arm, phase II innova204/GOG-3023/ENGOT-cx6 trial [86], 102 patients with squamous cell, adenocarcinoma, or adenosquamous cervical cancer previously treated with platinum-based chemotherapy and no more than two prior lines of therapy were enrolled. Patients received tisotumab vedotin at 2 mg/kg, up to a maximum of 200 mg, intravenously every 3 weeks until disease progression or unacceptable toxicity. The ORR was 24% (95% CI: 16–33), with 7 patients (7%) achieving a CR and 17 (17%) a PR. The median DoR, PFS, and OS were 8.3 (95% CI: 4.2–not reached), 4.2 (95% CI: 3.0–4.4), and 12.1 months (95% CI: 9.6–13.9), respectively [86]. Given the population of pre-treated patients for whom no standard of care is available, these results warrant further investigation. Main AEs were nausea (27%), conjunctivitis (26%), fatigue (26%), and dry eyes (23%). G3 toxicities were neutropenia (3%), fatigue (2%), ulcerative keratitis (2%), and peripheral neuropathy (2%). Interestingly, bleeding-related AEs occurred in 39% of patients, most of them G1 (34%) and the more frequent was epistaxis [86]. No significant changes in prothrombin time, international normalized ratio, or activated partial thromboplastin time were observed. The occurrence of this AE is more likely due to the elevated TF expression in the nasal epithelium [115] and not to a treatment-induced coagulopathy. No association between TF membrane expression levels and efficacy was demonstrated. However, most of the analyzed archival tumor samples showed a TF membrane expression > 1% with variable levels [86]. Thus, TF expression may contribute to the drug response, although additional mechanisms of action such as the bystander killing effect and immunogenic effects are involved. A phase III trial of tisotumab vedotin is ongoing to confirm its activity as a single agent compared to physician’s choice chemotherapy (NCT04697628).

The ENGOT-Cx8/GOG-3024/innovaTV 205 trials are investigating the efficacy of tisotumab vedotin in combination with carboplatin in first-line treatment of patients with recurrent/metastatic cervical cancer or in combination with pembrolizumab in the second or third line of treatment [87]. In the first-line setting, the combination of tisotumab vedotin and carboplatin resulted in an ORR of 55% and a mPFS of 9.5 months (4.0–not reached). In the second or third line, the combination of tisotumab vedotin and pembrolizumab showed an ORR of 38% and a mPFS of 5.6 months (2.7–13.7) [87]. These preliminary results support the continued investigation of tisotumab vedotin for the treatment of cervical cancer.

#### Anti-human trophoblast cell-surface marker: sacituzumab govitecan

Trop2 is highly expressed in cervical cancer tissue (88.7%), and its overexpression correlates with International Federation of Gynecology and Obstetrics (FIGO) stage, histological grade, lymphatic metastasis, depth of interstitial invasion, and high expression of Ki-67 [99]. As a consequence, patients with positive Trop2 expression had poorer OS and PFS [99].

In a preclinical study, the anti-Trop2 ADC sacituzumab govitecan was investigated on Trop2 positive cervical cancer cell lines and in xenograft models [116]. In this study, moderate to strong diffuse staining was seen in 95% of squamous cell carcinomas and in 81% of adenocarcinoma/adenosquamous subtypes. As previously described in endometrial cancers, Trop2 positive cell lines were highly sensitive to sacituzumab govitecan both *in vitro* and *in vivo* [116].

In the phase I/II basket trial of sacituzumab govitecan in patients with epithelial cancer only one patient with cervical cancer was included (efficacy data not shown) [72].

Main phase II and phase III studies of ADCs in relapsed or metastatic cervical cancer are summarized in (Tables 3 and 4).

## Mechanism of resistance to ADCs

As already described for many anticancer agents, innate or acquired resistance remains a major obstacle for the successful implementation of a treatment. Since ADCs are structurally complex molecules and their mechanism of action consists of several sequential steps, drug resistance might occur at different levels, as summarized below [117]. It is important to recognize that the main knowledge on the resistance to ADCs is available from the experience with sacituzumab govitecan [118] and T-DM1 [118–120], given the longer clinical experience with these drugs in breast cancer.

- Drug delivery: A pharmacokinetic mechanism explaining possible resistance to ADCs is the premature release of the payload from the antibody before it reaches the target cell. This event not only decreases the antitumor efficacy but also increases off-target systemic toxicity [117].
- Antibody-antigen binding: ADCs are highly dependent on the presence of a cellular antigen to exert their target effect. A first obstacle to the efficacy of an ADC may be the downregulation of the expression of target antigen on the cell surface and this might occur after chronic exposure to the drug [119].
- Drug catabolism: At the intracellular level, reduced lysosomal proteolytic activity can decrease the payload cleavage from the linker leading to a reduced cytotoxic effect [120].
- Mutation in the payload target: At the cellular level, possible mutations in genes encoding tubulin composition may also represent another mechanism of resistance to ADCs whose payload targets the mitotic spindle [121].
- Increased cellular drug clearance: Many payloads are substrates of the cellular efflux pumps [multidrug resistance protein 1 (MDR1) and multidrug resistance related protein 1 (MRP1)]. An increased expression of these transporters could reduce the intracellular concentration of cytotoxic molecules to ineffective levels [122].
- Tumor cells heterogeneity: Neoplasms with heterogeneous surface antigen expression might be unresponsive to ADCs that lack a “bystander killing effect”.
- Development of antidrug antibodies (ADA): ADA can develop against the different components of an ADCs (monoclonal antibody, cytotoxic payload, and linker). Their impact on ADCs safety and onset of resistance is under investigation [123, 124].

Different strategies are under investigation to overcome such mechanisms of resistance to improve ADCs efficacy [125]. A possible strategy to circumvent the drug clearance is to incorporate in the ADC a cytotoxic payload that is a poor efflux substrate. The payload of the ADC T-DXd, a topoisomerase I inhibitor deruxtecan (Dxd), is a poor substrate of the ATP-binding cassette (ABC) transporter that on contrary is involved in the resistance to T-DM1, due to the high affinity of DM1 to the efflux transporter. Thus, T-DXd has been shown to be active in overcoming the resistance to T-DM1 [126].

The heterogenous expression of the surface antigen might limit the ADCs efficacy, particularly in the low antigen-expressing cells. To overcome this limitation, an increase in the bystander effect is foreseen and this might be obtained by incorporating cytotoxic payloads whose charge allows an easy cell membrane crossing to reach the neighboring cells [65].

Another possibility to avoid or to overcome ADC resistance is the use of combination therapies and trials are ongoing investigating ADCs in association with chemotherapy or targeted agents. Another interesting and promising approach is the combination of ADC with immunotherapy [127].

## Conclusions

Despite the latest advancements and the introduction of targeted therapies, the prognosis of women with advanced/recurrent gynecological malignancies remains poor and new treatment options are urgently needed. Chemotherapy is usually effective in the first-line setting, but primary or acquired resistance inevitably occurs and subsequent treatment options are limited. The identification of novel agents with different mechanisms of action than standard cytotoxic chemotherapy is paramount to overcome drug resistance and to obtain disease control in subsequent lines of therapy. Furthermore, the use of chemotherapy is often limited by its toxicity profile, particularly hematological and neurological.

The development of ADCs offers the opportunity to selectively tackle the cancer cells expressing a particular target taking the advantage of the specificity of a monoclonal antibody to deliver potent cytotoxic agents. Despite the promise of reducing the toxicity through targeted delivery of the chemotherapy compound, different ADCs have shown significant off-target toxicities, due to the premature payload release into the circulation, the well-described ‘bystander effect’ and as a consequence of the expression of the target also in non-cancer cells. Since their first introduction, different generations of ADCs have been developed to improve their therapeutic index.

Different ADCs have been investigated for the treatment of women with gynecological malignancies and data from clinical trials, as presented in this review, are encouraging and support the continuous investigation of this innovative therapeutic strategy.

Among the drugs listed, mirvetuximab sorvatansine is the only one with available data from a phase III trial. In the FORWARD I study, the preliminary benefit observed in ORR, did not translate into a statistically significant prolongation of PFS [47]. Thus, it is necessary to wait for the results of other ongoing trials to define if this agent might represent a treatment opportunity particularly for patients with EOC and high FRα expression as a single agent or in combination.

The phase II, non-randomized study, innova204/GOG-3023/ENGOT-cx6 trial, showed both a PFS and OS benefit in patients with relapsed or metastatic cervical cancer treated with tisotumab vedotin [86]. Following the results of this trial, the FDA recently granted accelerated approval of tisotumab vedotin, for adult patients with recurrent or metastatic cervical cancer with disease progression during or after chemotherapy. Moreover, patients with non-squamous histology (adenocarcinoma or adenosquamous carcinomas), usually characterized by poor prognosis, were also included in this trial and responses were similar to those observed in the whole study population [86]. Considering the limited efficacy of the available therapies in recurrent/metastatic disease and the poor prognosis of patients with cervical cancer progressing after first-line treatment, the approval of tisotumab vedotin could be practice-changing in the treatment landscape of this disease, regardless of TF expression, histology, or prior treatment. Results from the ongoing phase III trial (NCT04697628) comparing tisotumab vedotin *versus* physician choice chemotherapy are awaited to confirm this preliminary data.

Preliminary signs of activity of ADCs have been seen also in endometrial cancer, particularly in the more aggressive histological subtypes. Despite the available multimodal treatment strategy incorporating surgery, radiotherapy, and chemotherapy, OS for patients with serous endometrial cancer varies between 18–27% [88]. Thus, the preclinical activity of sacituzumab govitecan and T-DM1 in this tumor type represents an important step forward [85, 89].

Finally, an increasing number of studies assessing the combination of ADCs and chemotherapy or other targeted agents including immunotherapy are ongoing.

Although there is still a lack of high-level evidence, this class of drugs has expanded the therapeutic possibilities in gynecological oncology with the final aim of improving patients’ outcomes and quality of life. Further research is still needed to define the best treatment regimen, predictive biomarkers and to understand the mechanisms of resistance. Particularly, a better definition of biomarkers with a strong predictive value is paramount for the clinical development of ADCs and their incorporation as a selection criterion in clinical trials is necessary to correctly exploit their efficacy, as clearly observed with mirvetuximab soravtansine in ovarian cancer. Moreover, incorporation of translational research particularly in early phase clinical trials is warranted to understand the mechanisms of primary or acquired resistance and improve the subsequent development of these agents for the treatment of gynecological malignancies, taking also advantage of the knowledge already established in other tumor types.

## Abbreviations

ADCs: antibody-drug conjugates
AEs: adverse events
CI: confidence interval
DAR: drug-to-antibody ratio
DM4: ravtansine
DoR: duration of response
EOC: epithelial ovarian cancer
FDA: Food and Drug Administration
FRα: folate receptor alfa
G: grade
HER2: human epidermal growth factor 2
IgG: immunoglobulin G
MMAE: monomethyl auristatine E
mPFS: median progression-free survival
MTD: maximum tolerated dose
MUC16: mucine 16
OS: overall survival
PFS: progression-free survival
PRs: partial responses
SD: stable disease
T-DM1: trastuzumab emtansine
T-DXd: trastuzumab deruxtecan
TF: tissue factor
Trop2: human trophoblast cell-surface marker 2

## Declarations

### Copyright

© The Author(s) 2022.
